# Equilibrium in the governance of cross-sectoral policies: how does it translate into practice?

**DOI:** 10.1186/s12961-023-01035-0

**Published:** 2023-09-13

**Authors:** Pernelle Smits, Johanne Préval, Jean-Louis Denis, Gerard Divay, Jacques Bourgeault, Nassera Touati

**Affiliations:** 1 Departement de Management, Université Laval, Montréal, Canada; 2 École Nationale d’administration Publique, CRCHUM, Montréal, Canada; 3 Université de Montréal, CRCHUM, Montréal, Canada

**Keywords:** Equilibrium, HiAP, Framework, Governance, Horizontal

## Abstract

**Background:**

There is growing interest from health researchers in the governance of Health in All Policies (HiAP). Furthermore, the COVID-19 pandemic has re-ignited managers’ interest in HiAP governance and in health prevention activities that involve actors from outside health ministries. Since the dynamics of these multi-actor, multi-sectoral policies are complex, the use of systems theory is a promising avenue toward understanding and improving HiAP governance. We focus on the concept of equilibrium within systems theory, especially as it points to the need to strike a balance between actors that goes beyond synergies or mimicry—a balance that is essential to HiAP governance.

**Method:**

We mobilized two sources of data to understand how the concept of equilibrium applies to HiAP governance. First, we reviewed the literature on existing frameworks for collaborative governance, both in general and for HiAP specifically, in order to extract equilibrium-related elements. Second, we conducted an in-depth case study over three years of an HiAP implemented in Quebec, Canada.

**Results:**

In total, we identified 12 equilibrium-related elements relevant to HiAP governance and related to knowledge, actors, learning, mindsets, sustainability, principles, coordination, funding and roles. The equilibria were both operational and conceptual in nature.

**Conclusions:**

We conclude that policy makers and policy implementers could benefit from mobilizing these 12 equilibrium-related elements to enhance HiAP governance. Evaluators of HiAP may also want to consider and integrate them into their governance assessments.

## Background

Health in All Policies (HiAP) refers to health policies that target a range of health determinants and involve multiple actors [1] and so are best administered using collaborative governance. Lessons learned about HiAP governance are drawn from two fields of literature: that on HiAP frameworks specifically, and that on collaborative governance in general.

There is a dearth of knowledge about HiAP governance, and what does exist revolves around the use of a step-by-step process to launch the implementation of the policy and oversee the intricate interlinking of actors to be mobilized. Shankardass et al. point out the importance of acknowledging the interconnections between the different subsystems at stake during HiAP implementation, namely, the executive, intra-sectoral and intersectoral subsystems [2]. Bilodeau et al. refer to chronologically ordered events, where the interplay of human and non-human entities transforms the web of actors and the health intervention itself into recomposed networks and modified living environments [3, 4]. Other studies have documented healthy cities and the involvement of multiple departments from health and non-health sectors under, for example, the direction of a mayor’s office or a health department [5].

Collaborative (or network or intersectoral or interorganizational) governance fits with the dynamics of HiAP. Indeed, it is understood as a network perspective whereby “…the multi-actor nature of interaction settings and the presence of diverging and sometimes conflicting perceptions, objectives, and institutions are the starting point for analysis and management” ([6] p14). At its heart, collaborative governance would seem to be an exercise in managing imbalances and tensions. Emphasis is placed on the multiplicity of actors, potential power/resource imbalances [7], unpredictable behaviours ([8] p335) and an open change process [2] that can react to disruptions [9]. We call the configuration of imbalances between different dimensions that leads to collective action an “equilibrium”.

We were interested in the concept of equilibrium in HiAP governance for several reasons. First, HiAP involves multiple actors, individuals and organizations, and calls for solutions to be found that benefit this collective work. It requires strengthening and taking into consideration the logic of each actor’s actions. We argue, as do others [10, 11], that embracing the apparent paradoxes behind network functioning is more informative than ignoring them, and that one needs to manage the structural ambidexterity of “hierarchy versus lateral relations, the existing power structure versus voluntary and involuntary power sharing” [12]. Second, a better understanding of the nature of the phenomenon of equilibrium will enable actors to, for example, better operationalize governance, define shared and complementary roles, and detect early signs of tensions. Overall, our goal is to better understand how HiAP governance is deployed and so offer guidance to managers who carry out HiAP governance, as well as promote discussion on the capacities needed to foster the achievement of equilibria. Finally, we argue for further research to explore equilibria in policy studies with a view to mobilizing theories on equilibrium management to explain policy-making outcomes.

Baumgartner and Jones’ punctuated equilibrium theory [13] discusses how to measure and explain the existence of long periods of policy-making stability and policy continuity that are disrupted by short but intense periods of instability and change during which there is a switch from one policy to another [14, 15]. Equilibrium is a concept that has caught the attention of many researchers, especially those interested in complexity theories. They have studied complex phenomenon using a variety of principles or concepts like chaos and adaptive systems, along with tenets like path dependence, system history, nonlinearity, emergence, irreducibility, adaptiveness, operating between order and chaos, self-organization [12, 16] and unstable chaotic systems.

Scholars in implementation science have studied equilibrium from the perspective of the policy/implementation nexus [17]. They argue that the implementation phase combines a single or combination of approaches, including prescriptive, desired, permitted, preconditioned or actual ones, in varying degrees of coherence.

In healthcare, the complexity of the sector makes it relevant to apply complexity theory because of its emphasis on agent interactions and emergent system outcomes [18]. Previous research has recommended equilibrium thinking in public health policy [19]. Generally speaking, in this research, equilibrium refers globally to a period of time in which there is a transition between stability and turbulence. By contrast, we examine the phenomenon of equilibrium as it applies to a policy’s period of stability, rather than to phase changes between a policy and a revised one, and place a greater focus on operational aspects. We focus on the concept of equilibrium, especially as it pertains to the need to strike a balance and feed a dynamic between actors involved in HiAP governance.

We mobilized two sources of data to understand how the concept of equilibrium applies to HiAP governance. First, we reviewed the literature on existing frameworks for collaborative governance, both in general and for HiAP specifically, in order to extract equilibrium-related elements. Second, we conducted an in-depth case study over three years of an HiAP implemented in in Quebec (Canada). The data that we collected during the implementation of this HiAP by policy makers highlighted the existence of contradictory dynamics that co-exist and co-evolve. We compared the elements revealed by the two separate analyses and then reconciled them with the five dimensions identified to come up with a final list of equilibrium-related elements applicable to HiAP governance.

## Step 1: Literature review on equilibrium in HiAP governance

### Methodology

We carried out a literature (scoping) review for frameworks that could be informative about equilibrium in the governance of multi-stakeholder and cross-sectoral policies like HiAP.

Scientific articles and grey literature from 2000 to 2019 were included. The search was carried out in Google Scholar, Google and the university database for topics related to aspects of equilibrium like trade-offs and related concepts (JSTOR, CAIRN). A combination of the following keywords was employed: collaborative/intersectoral/interministerial/joint/distributed/interstitial/boundary with governance/management/collective leadership, and policy/capacity/design/coherence/trade-off/input/output/synergy/adjustment/equilibrium/boundary/interstitial. Any document related to local initiatives and street-level managers was excluded. After a review of each title and summary, 66 documents were analyzed.

Equilibrium was broadly defined for this study as dynamics that could be in opposition and could potentially co-evolve, in other words as a state of oscillation between two extremes. This conceptualization is in line with how complexity theory conceptualizes complex adaptive systems that possess organizational capacities and move between order and transformational change [12]. Individual or organizational characteristics (e.g., skills, attitudes) or preliminary conditions for the governance of such policies were excluded, as equilibrium refers to dynamics in action. In total, we identified 21 documents that referred to equilibrium as defined above, 11 of which included frameworks (Table 1).

**Table 1:** Summary of frameworks that inform HiAP governance

First author, date	Name of framework	Equilibrium-related elements
Ansell, 2008	*A model of collaborative governance*	*Power-resource-knowledge asymmetries* *Informal work: trust building, commitment to process by mutual recognition of interdependence, shared ownership of process, openness to exploring mutual gain*
Purdy, 2012	*Power framework*	*Managing power imbalances*
Douglas, 2020	*Roadmap for achieving collaborative performance*	*Active interest alignment*
Bryson, 2006	*A framework for understanding cross-sector collaborations*	*Formal and informal process* *Formal and informal structure and governance* *Power imbalances* *Competing institutional logics*
Provan, 2008	*Three models of network governance forms*	*Management role to address tensions* *Tensions (contradictory logics) in each form* * • Efficiency versus inclusiveness* * • Internal versus external legitimacy* * • Flexibility versus stability*
Emerson, 2015	*Integrative framework for collaborative governance*	*Key driver of collaborative governance regimes: uncertainty about the nature of a given public problem and how to address it, and potential resources and future actions of others*
Greer, 2015 & 2019	*TAPIC: a governance framework to strengthen decision making and implementation*	*Ability to develop policy aligned with resources in pursuit of goals* *Opportunity for affected parties to provide inputs without fear of retribution*
McQueen, 2012	*Analytical framework for intersectoral governance*	*Shared evidence implies agreement upon acceptability of the evidence produced by all parties involved*
Berardo, 2016	*Shape of governance systems*	*Bonding structures* *Bridging structures*
Shankardass, 2018	*A system framework depicting 14 components from within three government sub-systems involved in HiAP implementation*	*Sub-systems in interactions* *Extra-governmental influences*
Bilodeau, 2018	*Systemic modelling based on Actor-Network theory*	*Aligning necessary actors and resources*

Text in italics is quoted directly from the source

### How the frameworks identified in the review conceive of equilibrium

In general, we found that existing frameworks for HiAP governance and collaborative governance do not directly consider equilibrium. Rather, they simply mention a concern for the management of balances either explicitly or indirectly. One framework mentioned how collaborative governance is affected by (im)balancing in representativeness among the participants, and how relationship building depends on formal and informal work [20]. Few studies referred to partners holding varied powers [21] and the management of power imbalances between cross-sectoral stakeholders [22]. As stated by Douglas et al. [23], the models of Bryson et al. [24], Ansell and Gash [21], Provan and Kenis [25] and Emerson and Nabatchi [26] seek to explain what makes collaborative governance work, for example, how a sense of interdependence among actors is created [21]. Other collaborative governance frameworks have been used to study and understand the implementation of interventions within cross-sectoral health governance [27, 28] and mention balanced rules, stakeholders and powers.

The frameworks dedicated to HiAP are silent on the topic of equilibrium (Table 1). There is discussion about the need to manage controversies in preventive public health interventions [3, 4]; to react to disruptions [29]; to overcome antagonisms [29]; to balance overt versus hidden agendas [29]; to overcome imbalances between win–win situations, neutral interests and more extreme situations of conflicts of interest [29]; and to manage the cross-influencing of subsystems [2].

### Equilibrium-related elements identified in the review

We identified six elements that pertain to equilibrium during governance in the literature.

#### Mindset: sectoral actions informed by a flow of multidisciplinary inputs

While at times information can be provided in a one-way information provision, collaborative development requires that attention be paid to the partners’ preferences about how they want to act strategically within the policy [30]. The complexity of public health policies, and the intended and unintended consequences of this, present major challenges and require that multidisciplinary knowledge be exchanged. For example, food security or levels of physical activity “emerge” from complex systems (socioeconomic, environmental, health promotion) and require the mobilization of data and evidence from different sources or disciplines [31]. The actions taken by each partner of a common HiAP are therefore fed by multiple inputs from various disciplines, which some partners may be used to integrating but others not. Balancing the flow of multidisciplinary inputs to inform HiAP implementation requires ongoing work. Collectively keeping track of decisions, progress and lessons learned is a potential challenge.

#### Knowledge: knowledge of bonding zones

For actors in governance, working together encompasses knowing where one stands in terms of individual and organizational knowledge on health and its determinants, knowing where partners stand in terms of this knowledge, and knowing how to bridge any gaps between the two. This requires that each participant is able to grasp the extent of the others’ knowledge and to either fill in the gaps or ensure brokering activities. Two attributes were emphasized in the literature: political knowledge, which is “information that an actor possesses about the policy preferences and strategies of other policy actors” and scientific knowledge, which refers to “actors’ perceptions of the adequacy of scientific understanding about the causes and consequences of the problems they face, and their possible solutions” [29, 32]. We also found that knowledge sometimes pertained to working together, such as individual and organizational competencies to work across boundaries, analyzing and involving stakeholders [33] and iterative learning [34]. A consensus on roles, tasks and missions [35, 36], as well as on what each partner understands about the policy with regard to the concepts of health and health determinants was widely accepted as a means to move forward collectively [3, 16, 37].

Overall, a knowledge of bonding zones, i.e., the potential for closeness, complementarities or similarities, comes from the knowledge of what the others know, do and are competent at doing. Such knowledge helps actors strike the right balance in collaborative HiAP governance and reach a modus vivendi between partners.

#### Actors: government accountability to implement a mandate with unbound extra-governmental actors

While HiAP implementation often mobilizes multiple government levels at the national, provincial/state, regional and/or local levels, and even extra-governmental systems [2] or intermediaries and mediators [3, 4, 22, 35, 38], the breadth of involvement can range from very few selected experts and representatives of organized groups to the general public [30]. It may even involve *tribal governments* and external organizations such as businesses, nongovernmental organizations (NGOs) and universities [39]. While the HiAP policy agenda frames each partner’s collective endeavour, coordination across various sectors of government and with extra-governmental partners is a balance that must be struck, one that includes actors at a similar horizontal level as well as those along different vertical lines.

#### Learning: seeking collective problem solving in the pursuit of a set agenda

A policy requiring multiple interventions from a wide range of organizational partners is subject to changes in partners’ priorities, which has ripple effects on the collective policy.

In such a situation, adaptive co-management is a form of governance that leaves room for the resilience and adaptability of systems to uncertainty and change [34, 40, 41]. Rules for co-solving approach have been set out [42]. In contrast to traditional command-and-control management, adaptive co-management with partners includes conflict resolution and a self-organized process of trial and error to move through the implementation stages of an interministerial action plan [34]. Co-solving problems creates fertile, and sometimes not so fertile, ground for further collaborations to move toward joint goals [43].

#### Principles: propagation of policy principles beyond the inner circle of partners

Organizations are committed to not just a single policy but to multiple collaborative activities [44]. Hence, social capital develops and grows stronger among the interacting organizations [45, 46], which may in turn generate more intensive and ongoing collaboration [39]. A consistent commitment by partner organizations for a health-related policy can be supported on a continuous basis in many ways: through advocacy to defend cross-sectoral work, by redirecting activities and focus areas of partner ministers closer to health, and through greater awareness of health prevention and social inequality in health [1, 38, 46–48]. One key dynamic pertains to keeping HiAP principles alive among partners, not only within the first circle of public servants who attend meetings, but also with their colleagues in charge of implementing other activities directly aligned with the organization’s agenda and also connected to health. Hence, creating an expanding wave of partners sensitive to the policy principles (e.g., prevention, equity) is key. It is a matter of establishing and actively maintaining the policy’s legitimacy with all levels of partners.

#### Sustainability of the policy topic: taking advantage of controlled entities for autonomous co-management

Partners all come with their own unique strengths, be it their knowledge of the problem, their partners onsite or their unique capacity to mobilize nongovernmental actors. Integrating the uniquely different resources of partners can facilitate the achievement of collaborative goals [10, 11, 49]. Each autonomous partner (without hierarchical link) delivers public services within the traditional vertical management of its hierarchy, yet also participates in horizontal work. Over time, balancing accountability and focusing on actions within the reach of horizontal objectives based on one’s own vertical strength could be a winning way to manage HiAP.

To summarize, our literature review of governance frameworks identified six equilibrium-related elements (see Table 3):

- Mindset: Sectoral actions informed by a flow of multidisciplinary inputs.
- Knowledge: Knowledge of bonding zones.
- Actors: Government accountability to implement a mandate with unbound extra-governmental actors.
- Mindset: Sectoral actions informed by a flow of multidisciplinary inputs.
- Learning: Seeking collective problem solving in the pursuit of a set agenda.
- Principles: Propagation of policy principles beyond the inner circle of partners.
- Sustainability of the policy topic: Taking advantage of controlled entities for autonomous co-management.

## Step 2: Equilibrium in the case study of HiAP governance

### Case description

Our empirical study concerned the implementation of a health promotion and disease prevention policy by the government of the province of Quebec (Canada). It involved actors located outside the health sector, and its goal was to promote/improve health and quality of life by acting on various elements, while also focusing on equity. The efforts targeted the reinforcement of support given to partners and the establishment of collaborations between partner organizations. The policy was led by the public health department of Quebec’s Ministry of Health and Social Services and relied on a cross-sectoral approach. The rationale was that it was important to involve a variety of stakeholders and actors in order to deal with societal changes that have, or will have, either direct or indirect repercussions on the population’s health and quality of life. A significant number of ministries and/or national organisms were involved throughout the process: a total of 15 partners (education, finance, agriculture, etc.) in addition to various other ministry of health (MoH) departments. For this policy, and in collaboration with its partners, the MoH elaborated an action plan with targets. As part of the governance process during the plan’s implementation, relevant bodies had to be put in place to anchor intersectoral governance at the decision-making and operational levels (Table 2).

**Table 2:** The case study: key organizations involved and their roles in HiAP governance

MoH (Ministry of Health)	• Provided leadership • Ensured all the necessary resources and conditions were in place to facilitate the implementation of the policy and the action plan • Monitored activities • Planned evaluations
Coordination bureau of MoH	• Coordinated • Directed the action plan implementation process, provided regular follow-up with the interministerial working group • Facilitated interministerial collaboration at all levels (intra-MoH, interministerial, and horizontal and vertical communication)
Partner organizations	• Implemented actions • Provided data to feed monitoring • Ensured direct contact with their usual local institutions, non-governmental organizations
Interministerial working groups (professionals, middle managers, top-ranked managers)	• Commented on future developments • Proposed adjustments • Validated new directions if needed (for top-ranked manager group)

We believe this case study is relevant for capturing the notion of multiple and intricated equilibria as it has a fair chance of being visible here for few reasons. Key activities that took place required some type of negotiations. This naturally created dynamics that could be in opposition and could potentially co-evolve, such as during the launch of the policy when each partner wanted some level of visibility, or during discussions about the allocation of additional funds to proposals by different partners, or when it came time to navigate the competing demands of daily administrative tasks and advocacy work aimed at newly elected officials.

### Methodology

Our data come from 58 secondary sources (e.g., reports, minutes), 16 semi-structured interviews with members of working groups and the coordination bureau, and 11 observations of meetings. The data were collected over a span of three years, namely from 2019 to 2021. A first analysis was carried out as part of a mandate to prepare a report for the lead ministry on governance and its success as a collective endeavor. A second analysis was intended to detect dynamics that could be in opposition and yet co-exist. Using a grounded theory methodology, two individuals reviewed the data and extracted all information related to the concept of equilibrium. They did not use any set categories for analysis initially so as to remain as open as possible in the search for equilibrium-related elements.

### Equilibrium-related elements identified in the case study

We identified two broad types of equilibria: conceptual equilibria, which are about ideas, and operational equilibria, which relate to tasks and daily work.

#### Mixing tangible and intangible aspects (conceptual equilibrium)

The HiAP initiative highlighted social inequalities in health. This involved launching awareness-raising activities for organizational partners on the policy from the point of view of social inequalities in health using a dedicated resource. However, the tangible application of social inequalities to the specifics of each sector was not achieved. At the same time, several of the organizational partners felt they were already working with vulnerable groups as part of their mission, thereby addressing social inequity even if the term “social inequity” was not being used. Thus, there was a gap between the tangible actions and intangible targets being pursued.

None of the partners mentioned any change in their way of approaching the plan’s implementation as compared to how they were used to implementing any other plan. This was despite brainstorming sessions being organized on the advantages and expectations of intersectoral management, and discussions being held on what aspects of intersectoral governance needed to be measured. While the lead ministry aimed to trigger new ways of working, this goal was not reflected in concrete terms. This points to tangible work being carried out without repercussions in the intangible realm.

In general, the HiAP governance observed in this case study alternated between ideas and their operationalization within the realities of the partner organizations, highlighting that an equilibrium has to struck between tangibles and intangibles. One is being accompanied by the other.

#### Fitting into an ecosystem of related plans (conceptual equilibrium)

The policy plan contained several measures, some of which were not new and were redundant with other sectoral policies. On the one hand, this created confusion among partners who were receiving instructions from different agencies responsible for multiple policy plans and generated a densification of accountability for wording changes that could be slight or marginal. On the other hand, this redundancy highlighted a certain level of consistency and linkages between interministerial plans within an ecosystem of plans. It allowed for continuity from one interministerial plan to the next, allowing for measures that had been planned but were not yet implemented to be kept in sight. Here, the policy and its instruments are seen to be positioned within an ecosystem of policies, both existing ones and those in the process of being made public. So the equilibrium is about fitting the policy within an ecosystem of already existing policies.

#### Operational equilibrium: a mix of formal and informal roles with decision makers

We observed the health policy’s governance during the funding phase, when interactions between administrators and policy makers were inevitable and were intended to feed policy makers. A concern for transmitting information in a concise and attractive format for policy makers marked this period. On the one hand, many notes to advise the minister of health were prepared and served to document the request for funding with explicit, easily interpretable figures. On the other hand, administrators demonstrated an ability to reach actors within the political sphere, including the health minister’s cabinet, and receive suggestions and advice from partners on the information expected by political actors. In this sense, we can see a mix of formal actions and informal networking at work.

#### Working with an acknowledged dissymmetry in the format of collective leadership (operational equilibrium)

As the many partners implemented the policy, the voices of all partners were heard at the discussion table. However, some partners lacked the necessary human resources or time to participate fully in the plan’s implementation. Since the implementation could not depend on each partner organization having the same capacities, and since the partner organizations did not have the same interests, issues and priorities as the MoH with respect to the HiAP, there had to be a balancing of capacities and organizational structures between the MoH and each partner organization.

#### Coordination of individual organizational capacities (operational equilibrium)

Consistent with the dissymmetry in resources and ways of handling organizational processes, including in the context of accountability, it was agreed to let each partner organization decide on what internal validation process to use for the accountability form.

As a result, collective reporting was done in coordination with a variety of ministerial management frameworks. One organization, for example, required that the reporting form be signed by several managers concerned by the various measures, while another organization only required a supervisor’s signature. Leeway was given for each organization to coordinate its own reporting internally using its usual processes. This points to a balance between the needs of the collective and the internal processes of partner organizations.

#### Sustainability of lessons learned (operational equilibrium)

The implementation of the plan was subject to seven evaluations. The usefulness of these for organizational learning and improving the subsequent plan was not discussed by the governance structures nor foreseen in any discussions underway as of March 2021. Subsequent to reporting, feeding back to partners on where they stood based on the data collected was to be the next step. However, the reporting tools were designed only for the lead ministry report; they did not take into account their usefulness or added value for the management of partner organizations. While reporting data were available, there was no plan to present the results in such a way that they could be useful to the partners, for example through specific support, data parties or infoletters. Overall, coordination was thin when it came to the development of innovations and new practices.

To summarize, our case study of HiAP governance identified six equilibrium-related elements (see Table 3):

- Mixing tangible and intangible aspects.
- Fitting into an ecosystem of related plans.
- Mix of formal and informal roles with decision makers.
- Working with an acknowledged dissymmetry in the format of collective leadership.
- Coordination of individual organizational capacities.
- Sustainability of lessons learned.

**Table 3:** The 12 equilibrium-related concepts for HiAP governance identified in this study, categorized by dimension

Literature review	Case study
Dimension 1: Degree of crossings between two types of knowledge	
Knowledge of bonding zones	Fitting into an ecosystem of related plans
Sectoral actions informed by a flow of multidisciplinary inputs	Working with an acknowledged dissymmetry in the format of collective leadership
Dimension 2: Combination of formal and informal relational positioning	
Government accountability to implement a mandate with unbound extra-governmental actors	Mix of formal and informal roles with policy makers
Dimension 3: Intensity of collective work to Do-it-Yourself practical solutions	
Seeking collective problem solving in the pursuit of a set agenda	Mixing tangible and intangible aspects
Dimension 4: Amplitude of kinetic effects from the collaborative governance	
Propagation of policy principles beyond the inner circle of partners	Sustainability of lessons learned
Dimension 5: Tolerance to variable engagement from partners, in their form and intensity	
Taking advantage of controlled entities for autonomous co-management	Coordination of individual organizational capacities

The first two elements relate to conceptual equilibria, while the other four relate to operational equilibria.

Overall, the literature on governance contains a great deal of information on equilibrium-related elements. We identified six key ones that address the aspects of knowledge, actors, learning, mindset, principles and sustainability. More specifically, the elements relate to the knowledge of bonding zones, sectoral actions that are informed by a flow of multidisciplinary inputs, government accountability to implement a mandate with unbound extra-governmental actors, seeking collective problem solving in the pursuit of a set agenda, the propagation of policy principles beyond the inner circle of partners, and taking advantage of controlled entities for autonomous co-management.

The case study was also very instructive in that it highlighted the need for HiAP coordinators to carry out tasks that are double-sided; to look not only inward to direct partners but also outward to final users; to seek not only a collective process but also make sure group leadership results in tools, mechanisms and means of communication; and to define roles clearly from the get go and at the same time leave leeway for informal roles to be occupied.

The complementary equilibrium-related elements uncovered in our two data sources—the literature review and the case study—are shown in Table 4. These elements pertain to all of the five main dimensions of HiAP governance shown in Table 3. We propose that the success of implementing such an HiAP depends upon the conjunctural equilibria between these five dimensions.

**Table 4:** Summary of the 12 equilibrium-related elements in HiAP governance identified in this study, categorized by source (literature review or case study)

Source	Element
Literature review	- Mindset: Sectoral actions informed by a flow of multidisciplinary inputs - Knowledge: Knowledge of bonding zones - Actors: Government accountability to implement a mandate with unbound extra-governmental actors - Learning: Seeking collective problem solving in the pursuit of a set agenda - Principles: Propagation of policy principles beyond the inner circle of partners - Sustainability of the policy topic: Taking advantage of controlled entities for autonomous co-management
Case study	- Mixing tangible and intangible aspects (conceptual equilibrium) - Fitting into an ecosystem of related plans (conceptual equilibrium) - Mix of formal and informal roles with policy makers (operational equilibrium) - Working with an acknowledged dissymmetry in the format of collective leadership (operational equilibrium) - Coordination of individual organizational capacities (operational equilibrium) - Sustainability of lessons learned (operational equilibrium)

## Discussion and conclusion

This study represents an important contribution to HiAP governance because it discusses in a detailed way what is meant by moving towards an equilibrium in such an initiative. Twelve items related to equilibrium are identified. These results are complementary to the broad frameworks dedicated to HiAP governance in which group and subgroup dynamics are given a strong analytical stance [2] and where key strategic categories for the national governance of HiAP are offered [47]. Here we point out the added value of looking at HiAP governance at the level of the managers responsible—not just at the point of deciding whether or not HiAP should be implemented, but rather after the decision is made and HiAP must be rolled out. Of particular interest would be further research on jurisdictions at different levels of maturity with respect to HiAP governance, and on how new insights into HiAP governance are similar to and different from the lessons we learned at national level.

This research has some limitations. Regarding the case study, it is worth noting that the equilibria were analyzed in a second wave of analysis, after focusing first on traditional aspects of governance like accountability mechanisms, communication tools, evaluation strategies and collective working mechanisms. The possibility of detecting equilibria by tracing artifacts is worth investigating further. The literature review covered only articles written in English, and the case study represents but one example and involved only actors at the provincial level. The list of equilibrium-related elements could have been longer had we conducted a multilingual search and/or examined an HiAP deployed at the local or municipal level. Any generalization of our results should be made with caution.

We decide to delve into the phenomenon of equilibrium because HiAP governance is a collective process. Underlying our interest was the assumption that governance would purposely seek a certain equilibrium, i.e., consider the 12 equilibrium-related elements listed, and perform better than one that does not. In a collective process that unfolds with a sense of achieving equilibria, governance would be more harmonious and lead to a stronger HiAP and to consistency in the multiple stakeholder actions aimed at health and well-being. The causal link between more consideration for equilibria in HiAP governance and better performance of the HiAP remains to be studied.

Additionally, policy studies and urban studies are paying increasing attention to the role of context in the study of HiAP. Analytical lenses such as the Walt and Gilson policy triangle framework, the fit-for-purpose framework and the theory of change of collaborative governance all refer to the role of context and its use in subsequently adapting implementation. With regard to HiAP in particular, the World Health Organization refers to the importance to getting to know the country context so that adjustments can be made to how the HiAP is implemented [50]. The results of the present research do not focus on any one specific variable in these frameworks, but rather serve to feed reflection on the connections between context and other aspects like actors, policy content, processes and outcomes. From this perspective, contextual elements appear in the form of, for example, shared problem solving to achieve policy outcomes.

It is our hope the present study responds to calls for more knowledge on the trade-offs that arise from policy that navigate ‘multiple (and often contradictory) objectives’ (51, p.259). It should also bring an added-value for future studies and frameworks on joined-up health policy governance and HiAP governance.

## Data Availability

The datasets generated and/or analyzed during this study are not publicly available due to restrictions imposed by the ethics committee, although some information may be obtained upon request by contacting the corresponding author.

## Abbreviations

HiAP: Health in all policies
MoH: Ministry of Health
